# cGMP favors the interaction between APP and BACE1 by inhibiting Rab5 GTPase activity

**DOI:** 10.1038/s41598-020-58476-8

**Published:** 2020-01-28

**Authors:** Francesca Caudano, Giulia Montalto, Mario Passalacqua, Maria A. Pronzato, Ernesto Fedele, Roberta Ricciarelli

**Affiliations:** 1 Department of Experimental Medicine, University of Genoa, Genoa, Italy; 2 Department of Pharmacy and Center of Excellence for Biomedical Research, University of Genoa, Genoa, Italy; 3 IRCCS Ospedale Policlinico San Martino, Genoa, Italy

**Keywords:** Cellular neuroscience, Molecular neuroscience

## Abstract

We previously demonstrated that cyclic guanosine monophosphate (cGMP) stimulates amyloid precursor protein (APP) and beta-secretase (BACE1) approximation in neuronal endo-lysosomal compartments, thus boosting the production of amyloid-β (Aβ) peptides and enhancing synaptic plasticity and memory. Here, we further investigated the mechanism by which cGMP regulates the subcellular localization of APP and BACE1, finding that the cyclic nucleotide inhibits the activity of Rab5, a small GTPase associated with the plasma membrane and early endosomes. Accordingly, we also found that expression of a dominant-negative Rab5 mutant increases both APP-BACE1 approximation and Aβ extracellular levels, therefore mimicking the effects induced by cGMP. These results reveal a functional correlation between the cGMP/Aβ pathway and the activity of Rab5 that may contribute to the understanding of Alzheimer’s disease pathophysiology.

## Introduction

Early endosomes represent the first station of internalization and processing of the Alzheimer’s disease (AD)-related amyloid-β precursor protein (APP). Intracellular processing of APP consists of a well-characterized proteolytic path that involves β- and γ-secretases and leads to the formation of amyloid-β (Aβ) peptides^1,2^. Alternatively, this amyloidogenic route is prevented when the first cleavage of APP is operated by α-secretase at the plasma membrane^3^.

Shedding of APP by the main β-secretase BACE1 takes place in early endosomes and factors that interfere with APP or BACE1 internalization have an impact on the production of Aβ^4,5^. However, APP and BACE1 do not colocalize at the plasma membrane and follow distinct routes of internalization^6,7^. In particular, APP reaches early endosomes via a clathrin-dependent pathway, whereas BACE1 follows a clathrin/caveolin-independent route controlled by the small GTPase Arf6^8^. Under normal conditions, early endosomes marked by Rab5, another small GTPase, represent the major site of APP processing by BACE1^9,10^, but the mechanism by which APP meets BACE1 in Rab5-positive endosomes is still largely unknown.

Our previous reports demonstrated that the enhancement of cyclic guanosine monophosphate (cGMP) in neuronal microdomains triggers the internalization of APP and the APP-BACE1 interaction in early endosomal compartments^11,12^. Additionally, this effect induced the production of Aβ peptides, which was instrumental to sustain hippocampal long-term potentiation and memory formation^12^.

In the present study, we further characterized the trafficking of APP and BACE1 under cGMP stimulation, finding that the cyclic nucleotide inhibits the activity of Rab5 by lowering its GTP-bound conformation. Our results are consistent with a growing body of data pointing to the dysregulation of specific Rab GTPases in AD. In particular, overactivation of Rab5 has been shown to cause endosome enlargement, one of the earliest pathological alterations observed in the brain of AD and Down syndrome patients^13^, whereas expression of a dominant-negative Rab5 mutant was found to reverse neuronal atrophy in *Drosophila*^14^.

Although the mechanism(s) leading to endosomal changes in AD remain to be fully elucidated, the results presented here provide new molecular details that may help to understand the roles of Rab5 and Aβ peptides in neurodegeneration.

## Materials and Methods

### Cell culture and treatments

Mouse Neuro-2a (N2a) cells were grown in 50% Dulbecco modified Eagle’s medium (DMEM), 50% OptiMEM, with 0.1 mM non-essential amino acids, 1% penicillin-streptomycin mixture, and 5% fetal bovine serum. Vardenafil and Pitstop2 (Sigma-Aldrich, Italy) were dissolved in dimethyl sulfoxide (DMSO) and stored at −20 °C until use.

### Plasmids and transfections

mCherry-Rab5^WT^ (expressing wild-type Rab5; Addgene plasmid # 14437) was a gift from Ari Helenius^15^; mCherry-Rab5^CA^ (expressing the constitutively active Rab5:Q79L mutant; Addgene plasmid # 35138) and mCherry-Rab5^DN^ (expressing the dominant negative Rab5:S34N mutant; Addgene plasmid # 35139) were a gift from Sergio Grinstein^16^. Plasmids encoding Arf6 proteins (HA-Arf6^WT^, HA-Arf6:Q67L, HA-Arf6:T27N) were kindly provided by Anna Fassio^17^. APP:VN (APP tagged with Venus N-terminal fragment) and BACE1:VC (BACE1 tagged with complementary Venus C-terminal fragment) plasmids were a generous gift from Subhojit Roy^18^. N2a cells were transiently transfected using Lipofectamine 2000 (Invitrogen, USA) at 2 μl/μg DNA.

### RNA interference

Accell Mouse Rab5a siRNA SMART pool and Accell Non-targeting siRNA Pool were purchased from Dharmacon (Lafayette, USA). Transfections were performed according to the manufacturer’s instructions and silencing efficiency was verified by immunoblotting.

### Aβ evaluation

ELISA kits (High Sensitive Human/Rat β Amyloid (42) and Human/Rat β Amyloid (40), Wako Chemicals GmbH, Germany) were used to measure Aβ peptides released into culture media from N2a cells. At the end of treatments, conditioned media were collected, spun at 1000 *g* for 5 min to remove cell debris, and stored at −80 °C until use. Were indicated, intracellular Aβ_42_ was measured in total cell extracts. ELISA tests were carried out following the manufacturer’s protocol, and the concentration of Aβ was calculated according to the standard curves prepared on the same ELISA plates.

### Rab5 activity

N2a cells were grown overnight on 10 cm culture dishes and then incubated with 100 μM vardenafil (or with the same volume of DMSO) for the indicated times. At the end of treatments, Rab5 activity was analyzed with the Rab5 Activation Assay Kit (NewEast Biosciences, USA), according to the manufacturer’s protocol. Briefly, an anti-active Rab5 mouse monoclonal antibody was used to immunoprecipitate the GTP-bound form of Rab5 in the cell extract. Immunoprecipitated Rab5 was then detected by immunoblot analysis using a rabbit anti-Rab5 polyclonal antibody.

### Immunoblot analysis

Total protein extraction from cell cultures and immunoblots were performed according to standard methods, as described previously^19^. Anti-rabbit and anti-mouse secondary antibodies were coupled to horseradish peroxidase (GE Healthcare, UK). Protein were visualized with an enzyme-linked chemiluminescence detection kit according to the manufacturer’s instructions (Amersham, UK). Chemiluminescence was monitored by exposure to films, and signals were analyzed under non-saturating condition with an image densitometer (Bio-Rad, USA).

### Confocal analysis

N2a cells were grown overnight on culture slides and transiently transfected with mCherry-Rab5 mutants where indicated. After 16 hours, cells were transfected with APP:VN and BACE1:VC expressing vectors and, 6 hours later, incubated with DMSO or vardenafil (50 μM) for 16 hours. At the end of treatments, cells were permeabilized and fixed with ice-cold methanol, incubated with TO-PRO-3 Iodide (Thermo Fisher Scientific, Italy) for nuclear staining, and observed with the appropriated filters on a Leica TCS SP2 confocal microscope (planapochromat x 60 oil-immersion objective, numerical aperture 1.4).

### Statistical analysis

Results are expressed as mean ± standard error of the mean (SEM). The number of independent experiments is reported in each figure legends. Data were analyzed using one-away ANOVA followed by Dunnet’s post-hoc. The level of significance was set at *P* < 0.05.

## Results

### Inhibition of endocytosis prevents the amyloidogenic effect of cGMP

**Figure 1:**
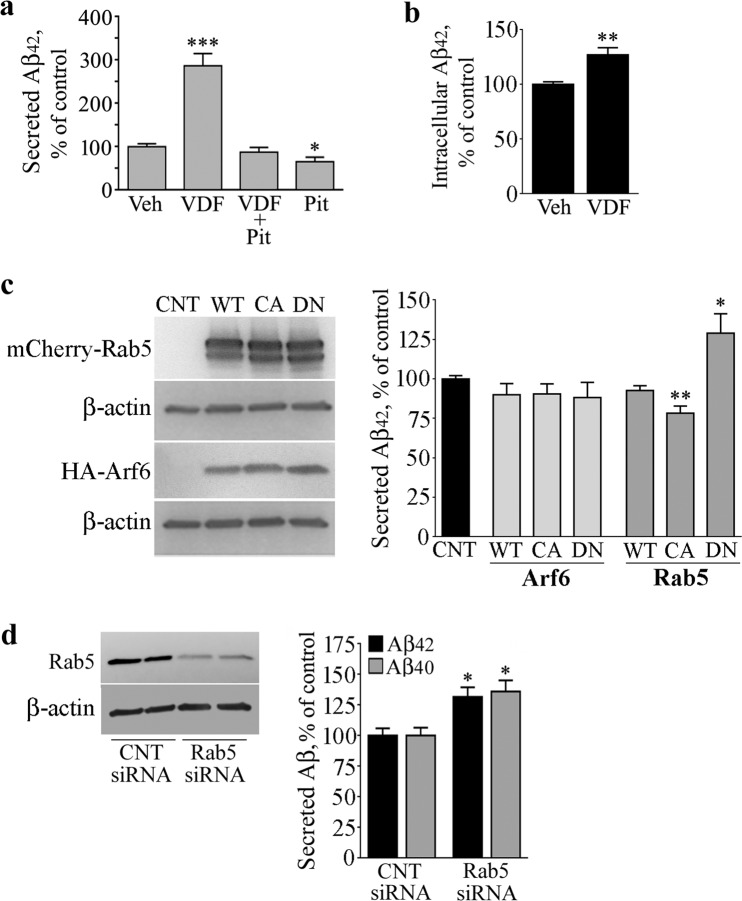
Inhibition of Rab5 increases Aβ peptides. (**a**) To verify the role of endocytosis in the vardenfil (VDF)-stimulated Aβ_42_ release, N2a cells were pretreated with 25 μM PITSTOP2 for 10 min and then incubated for 1 h with 100 μM VDF or an equal volume of vehicle (DMSO). At the end of the incubation period, conditioned media were subjected to specific Aβ_42_ ELISA. Graphed data show mean ± SEM of four independent experiments (*P < 0.05; ***P < 0.0001 vs the vehicle-treated group). (**b**) N2a cells treated with 100 μM VDF or vehicle for 1 h were processed for total protein extraction. Protein extracts were then subjected to Aβ_40_ ELISA. Graphed data show mean ± SEM of four independent experiments (**P < 0.01 vs vehicle-treated group). (**c**) Where indicated, N2a cells were transfected with HA-tagged Arf6 (WT), HA-Arf6 Q67L (CA), HA-Arf6 T27N (DN), or with mCherry-Rab5 (WT), mCherry-Rab5 Q79L (CA), and mCherry-Rab5 S34N (DN). After 24 h, conditioned media were subjected to specific Aβ_42_ ELISA, while cells were processed for total protein extraction followed by immunoblot analysis performed with anti-Rab5 and anti-HA antibodies. The β-actin signal represents the internal loading control. The image shows cropped blots and is representative of three independent experiments; graphed data show mean ± SEM (*P < 0.05; **P < 0.005 vs control group). (**d**) N2a cells were transfected with Rab5 siRNA or non-targeting siRNA (CNT siRNA). After 48 h, media were changed and collected 1 h later for Aβ_40_ and Aβ_42_ ELISA. At the same time, cells were processed for Rab5 immunoblotting. The β-actin signal represents the internal loading control. The image shows cropped blots and is representative of three independent experiments; graphed data show mean ± SEM. (*P < 0.05 vs corresponding control).

Since our recent studies showed that cGMP induces a faster internalization of APP and triggers APP and BACE1 to colocalize in endosomal compartments^11,12^, we first investigated the impact of endocytosis on the cGMP-induced amyloidogenesis. To this aim, N2a cells were pre-treated with PitStop2, an inhibitor of both clathrin-dependent and clathrin-independent endocytic pathways^20^, and then exposed to the cGMP-enhancer vardenafil. As expected, the inhibition of endocytosis *per se* reduced the concentration of Aβ_42_ peptides in conditioned media^4^ (67% of control, P < 0.05), whereas vardenafil robustly increased it^11^ (286% of control, P < 0.0001). Notably, the effect of vardenafil was totally prevented by PitStop2, indicating that the endocytic process is required for cGMP to increase extracellular Aβ_42_ (Fig. 1a). In addition, also intracellular levels of Aβ_42_ were significantly increased under vardenafil treatment, though to a lesser extent compared with the secreted peptide (130% of control, P < 0.01), providing further support to the evidence that cGMP plays a role in the processing of APP (Fig. 1b).

### Rab5 activation state modulates Aβ42 levels

Given the involvement of Rab5^14^ and Arf6^8^ in APP and BACE1 endosomal trafficking, respectively, we evaluated the amount of Aβ_42_ peptides in conditioned media of cells transiently transfected with mCherry-Rab5^WT^, HA-Arf6^WT^, or their constitutively active (mCherry-Rab5^CA^, HA-Arf6^CA^) and dominant-negative (mCherry-Rab5^DN^, HA-Arf6^DN^) mutants. Efficiency of transfections in multiple experiments was assessed by immunoblot analyses, and typical levels of expression are shown in Fig. 1c (left panels). Overexpression of either WT or mutant forms of HA-Arf6 did not alter the amount of Aβ_42_ released by the cells in the culture media, and similar results were obtained in mCherry-Rab5^WT^ expressing samples (see the histogram in Fig. 1c). On the contrary, overexpression of mCherry-Rab5^DN^ increased the Aβ_42_ release (130% of control, P < 0.05), while a slight, but statistically significant decrease was observed in culture media of cells transfected with mCherry-Rab5^CA^ (83% of control, P < 0.01) (see the histogram in Fig. 1c).

### Rab5 siRNA increases both Aβ_42_ and Aβ_40_ peptides

In order to confirm the correlation between Rab5 and Aβ production, we induced Rab5 transient knockdown in N2a cells using specific siRNAs. Forty-eight hours after siRNA transfections, conditioned media were subjected to Aβ specific ELISA, whereas cell extracts were analyzed for Rab5 expression by immunoblot. In line with the effect exerted by Rab5^DN^, silencing of Rab5 significantly increased the amount of Aβ_42_ released by the cells in the culture medium (131% of non-targeting control siRNA, P < 0.05) (Fig. 1d). Of note, also the Aβ_40_ species increased in the medium of Rab5 knockdown cells (136% of non-targeting control siRNA, P < 0.05), indicating that the amyloidogenic effect of Rab5 siRNA does not change the Aβ_40_/Aβ_42_ ratio (Fig. 1d).

### cGMP reduces GTP-Rab5 levels

**Figure 2:**
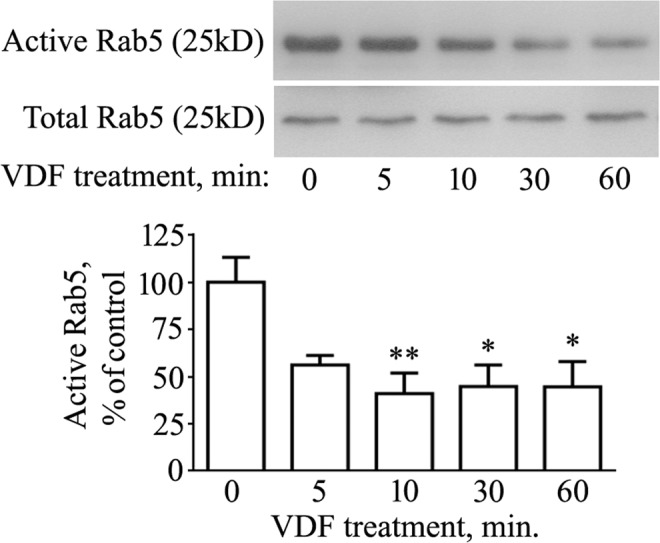
The cGMP enhancer vardenafil reduces active Rab5 levels. N2a cells were treated with 100 μM vardenafil (VDF) for different times (5, 10, 30, 60 min). Control samples (0 min) received the same volume of vehicle (DMSO) for 60 min. At the end of treatments, cells were processed for total protein extraction followed by Rab5 activity assay, as described in the Methods section. The image displays cropped blots. Graphed data show mean ± SEM for four independent experiments (*P < 0.05; **P < 0.005 vs the vehicle-treated group).

We next sought to examine whether an increase of intracellular cGMP could influence the activation of Rab5. As a small GTPase, Rab5 cycles between a GDP- (inactive) and a GTP-bound form (active)^21^. Therefore, we measured GTP-Rab5 levels in cells exposed to vardenafil for different times (5, 10, 30, and 60 min). As shown in Fig. 2, an approximately 50% reduction of active GTP-bound Rab5 was already evident, although not statistically significant, after 5 min of vardenafil treatment (P = 0.088 vs control). The amount of GTP-Rab5 dropped to 40% of control after 10 min (P < 0.01) and remained depressed after 30 and 60 min of treatment (45% of control, P < 0.05, at both time points). Because the total expression of Rab5 did not change at any time of vardenafil exposure (Fig. 2, Total Rab5 panel), it is likely to assume that cGMP increased the inactive GDP-bound form of the small GTPase.

### Expression of Rab5^DN^ mutant increases APP-BACE1 approximation

**Figure 3:**
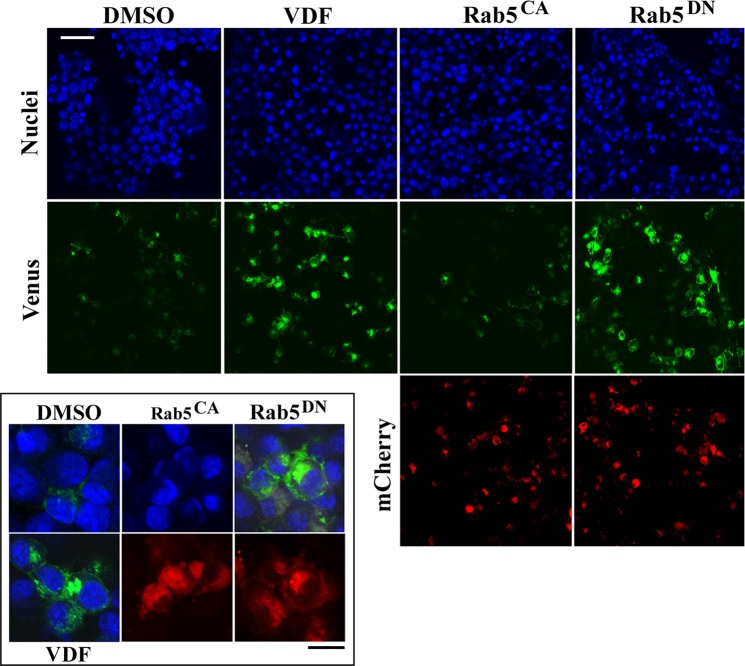
Rab5^DN^ mutant triggers the interaction between APP and BACE1. For confocal analyses, N2a cells were transfected with APP:VN and BACE1:VC expressing vectors. Only where indicated, cells additionally expressed mCherry-Rab5^DN^ (Rab5^DN^), mCherry-Rab5^CA^ (Rab5^CA^), or underwent vardenafil treatment (VDF). Nuclei are blue-fluorescent, the green fluorescence is indicative of APP-BACE1 interaction, whereas the red fluorescence is due to the expression of mutant mCherry-Rab5. Note the increase in the APP-BACE interaction in cells treated with vardenafil compared to control cells and how this effect is mimicked by Rab5^DN^ but not by Rab5^CA^ expression. The inset shows merged blue/green fluorescence, and mCherryRab5 expression, at a greater magnification. White scale bar = 50 μm; black scale bar = 20 μm. Figure is representative of 3 independent experiments, all showing essentially similar results.

Using the Optical Convergence of APP and BACE1 (OptiCAB) assay^18^, we have previously demonstrated that cGMP stimulates the interaction between APP and BACE1 in endosomal compartments^12^. Here, given the effect of vardenafil on the activation state of Rab5, we took advantage of the OptiCAB assay to investigate whether the expression of Rab5 mutants could affect the APP-BACE1 approximation. To this aim, 16 hours after mCherry-Rab5^DN^ or mCherry Rab^CA^ transfections, N2a cells were further transfected with APP tagged with the N-terminal fragment of the Venus fluorescent protein (APP:VN), and with BACE1 tagged with the complementary C-terminus (BACE1:VC). In this manner, the physical approximation of APP:VN and BACE1:VC allows the reconstitution of Venus protein, which becomes fluorescent. Indeed, confocal microscopy analysis showed that the expression of Rab5^DN^ is able to increase APP-BACE1 approximation (Fig. 3), which is suggestive of an increased interaction between BACE1 and its substrate. Notably, this effect exactly resembled that induced by the cGMP-enhancer vardenafil and was not mimicked by the expression of Rab5^CA^ (Fig. 3).

## Discussion

In this study, we provide evidence that cGMP influences the activation state of Rab5 by favoring its inactive GDP-bound conformation. This conclusion is supported by a coherent set of data. Firstly, the amount of Rab5 bound to GTP is quickly reduced by vardenafil, a phosphodiesterase 5 (PDE5) inhibitor whose ability to enhance cGMP in N2a cells is widely documented^12^. Secondly, expression of a dominant mutant that keeps Rab5 in its GDP-bound form increases the amount of Aβ peptides released by the cells, in line with previous studies showing that cGMP stimulates Aβ production in a dose-dependent manner^11,12^. Thirdly, extracellular Aβ levels are increased when Rab5 expression is knocked down by siRNA. Finally, expression of the inactive Rab5 mutant increases APP-BACE1 interaction, an effect previously observed in primary neurons treated with vardenafil^12^, and further confirmed in the present study.

Taken together, these results suggest that the well-established positive effects exerted by cGMP on synaptic plasticity and memory formation (for review, see^22^) may occur via lowering Rab5-GTP levels, thus explaining, at least in part, why the upregulation of the small GTPase is associated with neurodegenerative phenomena^13^.

Another important evidence in the present study is that the inactivation of Rab5 correlates with an increased production of Aβ peptides, an event that, based on the “amyloid hypothesis” of AD^23^, would generate detrimental consequences. Indeed, our data fit well with the notion that released Aβ boosts hippocampal activity by regulating synaptic trsnsmission^24^, and with the studies of Puzzo and collaborators, who clearly demonstrated that physiological (picomolar) concentrations of extracellular Aβ_42_ are required to sustain LTP and cognitive performance^25,26^.

On the other hand, our findings seem at variance with previous reports showing that overexpression of either wild-type Rab5^27^ or dominant negative Arf6-T27N^8^ increases the production of Aβ peptides. It should be noted, however, that those studies have been performed in non-neuronal cells engineered to overproduce Aβ, thus under different conditions that might justify the different results.

Consistent with this hypothesis, a new transgenic mouse model of neuronal Rab5 over-activation was very recently shown to develop AD-related endosome dysfunction and AD-like deficits in axonal transport, synaptic plasticity, cognition, and neuron survival, apparently without increasing cerebral Aβ. Noteworthy, crossing this Rab5 model with APP-models of AD accelerated Aβ accumulation and cholinergic neurodegeneration, suggesting that APP mutation/elevation may change the effect of Rab5 activation on Aβ production (Nixon, R. A. Rab5 hyper-activation and aberrant endosomal signaling: a new mouse model of early stage Alzheimer’s Disease. 14th International Conference on Alzheimer’s & Parkinson’s Diseases. 26–31 March 2019, Lisbon, Portugal).

Collectively, our data support a scenario in which cGMP favors the endosomal interaction between APP and BACE1 by keeping Rab5 in its GDP-bound conformation, consequently leading to the production of Aβ peptides that, in turn, sustain LTP and memory formation/consolidation^12,26^.

Although we are still far away from elucidating the complete picture, novel molecular players governing the dynamics of Aβ production are beginning to emerge. Certainly, the more we learn on the physiology of APP and its processing, the closer we get to understanding AD and finding effective pharmacological treatments.

From this prospective, increasing cGMP levels with PDE5 inhibitors could represent a promising therapeutic strategy. As a matter of fact, a study investigating repeated administrations of udenafil in patients suffering from erectile dysfunction has shown beneficial effects on memory performance as well as on a battery of tests assessing executive functions^28^. For a more complete information, however, it must be said that, in healthy subjects, single vardenafil administration did not produce cognitive or information processing modifications^29^.

## Data Availability

The authors make materials, data and protocols associated to this article promptly available to readers without undue qualifications in material transfer agreements.
